# Bilateral Circumscribed Posterior Keratoconus: Visualization by Ultrasound Biomicroscopy and Slit-Scanning Topography Analysis

**DOI:** 10.1155/2012/587075

**Published:** 2012-02-13

**Authors:** Robert Rejdak, Katarzyna Nowomiejska, Dariusz Haszcz, Anselm G. M. Jünemann

**Affiliations:** ^1^Department of Ophthalmology, Medical University of Lublin, 20-059 Lublin, Poland; ^2^Department of Ophthalmology, University of Erlangen-Nürnberg, 91054 Erlangen, Germany

## Abstract

This paper documents a rare nonprogressive developmental disorder—bilateral circumscribed posterior keratoconus—in a 60-year-old man referred for a cataract surgery. For the first time ultrasound biomicroscopy was used to visualise the local anterior bulging of the posterior corneal surface with concomitant thinning of the stroma. The amount of localized posterior depression, corneal thickness and the refractive power of both the posterior and anterior corneal curvature were measured using slit-scanning topography analysis (Orbscan).

## 1. Introduction

Abnormal variation of the posterior corneal curvature may occur in two forms: the generalized posterior keratoconus, characterized by an regular increase of the curvature of the entire posterior corneal surface has, and the circumscribed posterior keratoconus, in which a localized paracentral or central posterior corneal indentation is seen [1]. In the generalized form, the corneal stroma typically remains clear. In contrast, the circumscribed posterior keratoconus shows stromal opacities overlying the localized anterior ectasia of the posterior surface, which may occupy the full stromal thickness [2]. The visual loss is not progressive and moderate [3]. Vision deterioration usually is caused by corneal scarring or amblyopia. Circumscribed posterior keratoconus is usually bilateral and sporadic, but familial cases have been also documented [4]. Despite the anterior protrusion in some cases, posterior keratoconus does not progress to anterior keratoconus and normally requires no treatment. Usually it is detected during routine ophthalmic examination. We describe a case of bilateral posterior circumscribed keratoconus.

## 2. Case Report

The 60-year-old white male of Mediterranean origin presented for a cataract extraction on his left eye. Visual acuity was 20/25 in the right eye and light perception in the left eye due to cataract formation. There was no amblyopia in the left eye before the onset of cataract. The patient denied history of injury, reporting only a bilateral ocular infection in childhood was reported. There were no systemic conditions.

**Figure 1:**
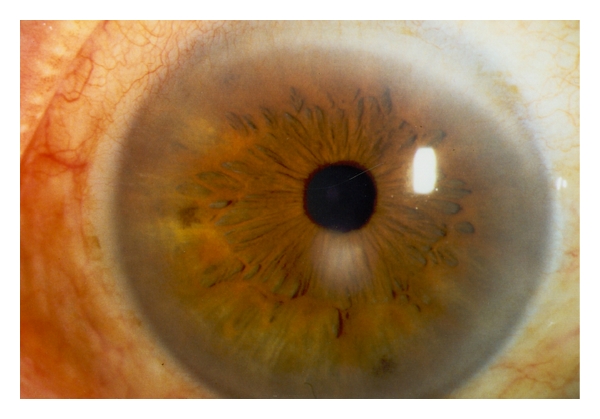
Right eye showing paracentrally inferiorly circumscribed corneal opacification.

**Figure 2:**
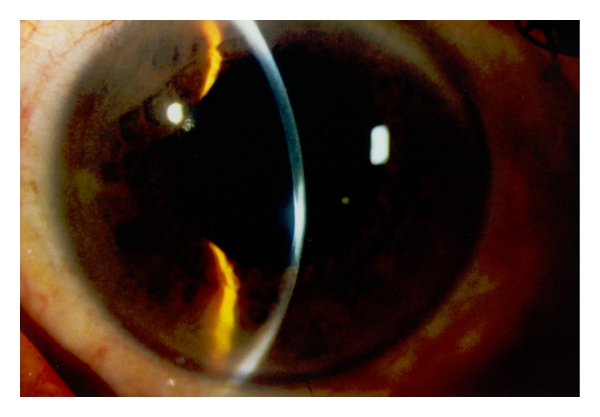
Slitlamp photograph showing circumscribed protrusion of the posterior corneal curvature with concomitant stromal thinning and an opacification of the overlying stroma.

**Figure 3:**
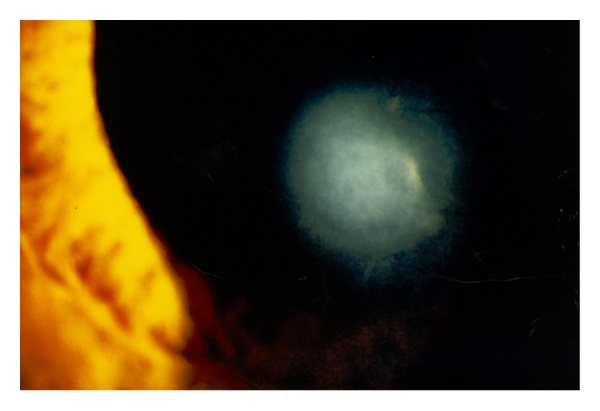
High magnification shows a relatively dense opacification of the cornea. Note the retrocorneal melanin granules at the edge of the stromal opacity.

**Figure 4:**
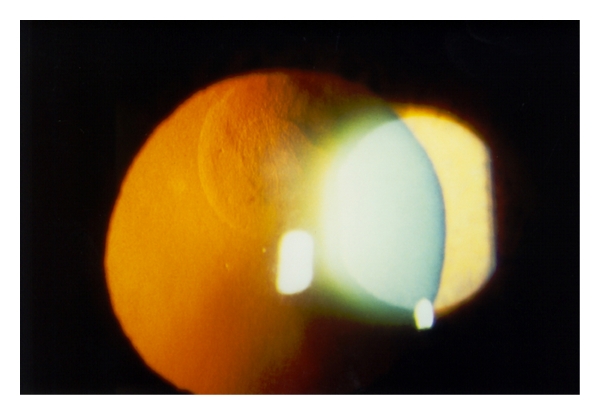
Retroillumination shows an irregularity with mosaic-like pattern. Note the sharp margin of the round lesion (arrow). There is a second sharp round line (arrowhead), forming a central and a peripheral zones.

**Figure 5:**
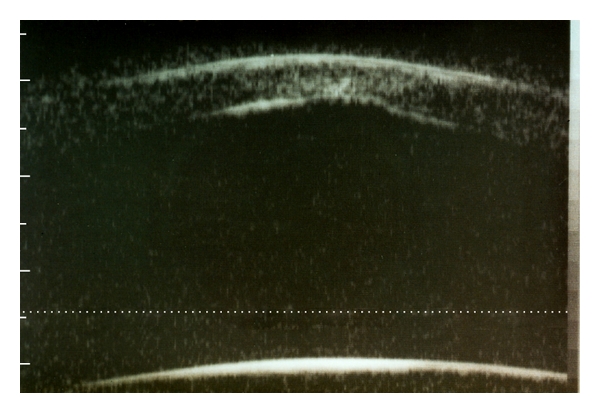
Ultrasound biomicroscopy shows the local anterior bulging of the posterior corneal surface with concomitant thinning of the stroma. Note the configuration of the enhanced stromal reflectivity (arrowhead) corresponding to the stromal opacity.

**Figure 6:**
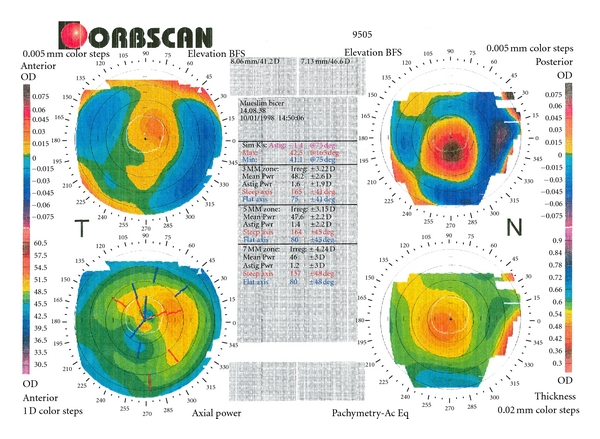
Slit-scanning topography analysis of the posterior corneal curvature (Orbscan) shows the circumscribed protrusion of the posterior surface, located paracentrally inferiorly. The color code indicates a “bulging” of about 75 *μ*m.

Slitlamp examination revealed a bilateral paracentrally localized depression of the posterior curvature measuring 3 mm in diameter. There was scarring in the overlying corneal stroma (Figures 1, 2, and 3). An intraepithelial iron line was noted at the base of the lesion temporally. A few retrocorneal melanin granules were present (Figure 3). An irregular mosaic-like pattern was noted using retroillumination (Figure 4). The posterior depression was clearly detectable using ultrasound biomicroscopy (Humphrey, Zeiss, Oberkochen) (Figure 5) and slit-scanning topography analysis (Orbscan, Bausch and Lomb) (Figure 6). The amount of localized posterior depression was 75 *μ*m as indicated by topography. Corneal thickness measured 450 *μ*m within the lesion and 540 *μ*m in the adjacent healthy cornea using the Orbscan system. The refractive power of both the posterior and anterior corneal curvature was 50 to 56 diopters within the paracentral area. Otherwise, both eyes were unremarkable. Following phacoemulsification and posterior chamber lens implantation visual acuity increased to 20/50 in the left eye. The examination of the fundus and vitreous revealed no pathological findings.

## 3. Discussion

The clinical and topographic findings in this patient are consistent with the *paracentral keratoconus posterior circumscriptus *[5]. This is the first report on ultrasound biomicroscopy to visualise the local anterior bulging of the posterior corneal surface with concomitant thinning of the stroma. Light microscopy of this abnormality has shown focal disorganization of basal epithelium and basement membrane, a replacement of Bowman's layer by fibrous tissue, a thinned stroma with an irregular arrangement of the central collagen lamellae, and a variable appearance of Descemet's membrane [6] with posterior excrescences indentating the vacuolated endothelium correspond to the corneal guttae seen in specular reflection [7]. Iron deposits are present in the basal and suprabasal epithelium, corresponding to the brownish epithelial line observed clinically [7], indicating an irregularity of the anterior corneal surface. Visualisation of the posterior keratoconus using corneal topography analysis has been reported so far in a few cases [7, 8].

The condition is thought to be a developmental disorder. The light microscopy findings suggest an early pathogenic mechanism probably originated in the fifth or sixth month of gestation [6]. It is classified as one of the anterior chamber cleavage anomalies (mesenchymal dysgenesis), as there are other anterior segment and systemic developmental abnormalities, as well as melanin depositions surrounding the posterior depression and iridocorneal adhesions [7]. However, not all cases share this phenomenon. Acquired cases occur and are usually associated with trauma [9, 10]. The mechanism in such cases involves an oblique penetrating injury with splitting of the inner corneal layers. Differential diagnosis also includes congenital disorders as Peter's anomaly and congenital hereditary endothelial dystrophy but they are usually found in new borns. Inflammation process as perforated corneal ulcer may also be taken into consideration, but it is usually unilateral. In most of the cases of posterior keratoconus the vision is not affected, rarely it may be associated with other ocular abnormalities as polar cataract, lenticonus, and ectopia lentis.
